# Display consistency in swallow-tailed manakins: group size effects and implications for female choice

**DOI:** 10.1098/rsos.250943

**Published:** 2025-10-29

**Authors:** Pedro Henrique L. Ribeiro, Sabrina B. L. Araujo, Thiago L. Prado, Sergio R. Lopes, Lilian T. Manica

**Affiliations:** ^1^ Graduate Program in Zoology, Universidade Federal do Paraná, Curitiba, Brazil; ^2^ Department of Physics, Universidade Federal do Paraná, Curitiba, Brazil; ^3^ Department of Zoology, Universidade Federal do Paraná, Curitiba, Brazil

**Keywords:** female choice, predictability, recurrence quantification analysis, sexual selection, time series

## Abstract

Courtship displays indicate individual quality and can direct mate choice. Cooperative displays are particularly interesting because the choice can be complex, involving the evaluation of multiple individuals’ coordination. Here, we characterize the consistency of the cooperative display of the swallow-tailed manakin (*Chiroxiphia caudata*), in which males aggregate in courtship groups of up to six individuals. We generated a time series of a set of displays and calculated its temporal frequency, number of males and consistency. We applied the recurrence quantification analysis to characterize consistency in terms of two metrics that describe the overall pattern—recurrence rate and determinism—and one metric that detects subtle changes on the display—microstate entropy. We assumed that consistency increases as the first two measures increase and the latter decreases. Our results revealed that microstate entropy was the only metric sensitive to group size, suggesting that males reduce individual energy expenditure through cooperative displays. Despite the dance’s overall stability across group sizes, females preferentially visited and copulated with males performing highly consistent displays (higher recurrence rate, lower microstate entropy). This demonstrates that fine-scale temporal precision—rather than display frequency or group size—drives female choice, implicating consistency as a target of sexual selection in swallow-tailed manakins.

## 1. Introduction

Sexual selection explains the evolution of elaborate displays, which can signal health conditions and influence mate choice [1]. In birds, displays can be visual (as the colourful feathers of birds of paradise [2]), auditory (as the complex song of thrushes [3]) or both, in which males dance and sing during their acrobatic performances (as in the Neotropical manakins [4,5]). Even more complex is the cooperative display involving several males, an unusual behaviour among animals, found mostly among birds and fish [6] and invertebrates [7]. Males can join and cooperatively display to a female for a variety of reasons; for example, because it increases their reproductive success through enhanced competitive abilities against rivals or their sexual attributes [6]. However, studies with cooperation in the reproductive context are not abundant, with a lack of information on the influence of sexual selection [6].

In cooperative systems, mate choice can be complex and based upon the group phenotype reflecting their consistencies (e.g. [8,9]). Consistency can be interpreted in terms of the ability of individuals to perform repetitive or standardized displays in a similar pattern of ordering and structure, considering their element components [10,11]. Sexual selection may have favoured consistent courtship displays within species since it increases the efficiency in communication, as it may impact signal perception and evaluation by receivers, facilitating species recognition [12], sexual stimulation and the mate choice process [13]. In multimodal displays, for example, the integration and coordination of several signal modalities will elicit a high demand for perceptual and interpretation ability of receivers [13,14]. Thus, display consistency may impact its function. In manakin species with dual-male displays, females are more attracted to those pairs performing well-matched songs in terms of frequency [8] and more consistent dances [9]. Furthermore, display quality may depend on the social context, such as pair formation length. For instance, males of long-tailed manakins (*Chiroxiphia linearis*) in a long-term partnership have a greater frequency matching in acoustic display [15]. In fish and invertebrates, the consistency of displays is strongly linked to group size and is hampered as the number of participating individuals increases [16,17]. This consistency has probably evolved because it increases the group’s visibility and the efficiency of communication [7].

A fundamental metric to characterize and quantify display consistency is the time frequency (the number of times a given element of the display occurs within a time window). Despite being an easy-to-interpret metric, it is usually insufficient since it neglects the temporal correlation between elements. As complementary characterization, other metrics have been used in several taxa (crustaceans: [18,19], birds: [9,10,20–22] and mammals: [23]). Recently, Perez *et al*. [24] used recurrence quantification analysis (RQA [25]) to characterize the crab display. Recurrence is a key property of dynamical systems, allowing the generation of time-complexity metrics to quantify and visualize the behaviour of a time series, i.e. a sequential set of data points measured at successive times [25]. RQA is composed of several metrics that together can identify not only the overall pattern (as recurrence rate (RR), determinism (DET) and laminarity (LAM)) but also subtle details of the time series (as microstate entropy, MCEntr [26]). Although RQA is not a widely applied analysis in display characterization, it is a powerful and consolidated method to characterize other time series. It has been mostly used to analyse physiological systems [27], non-stationary cardiac signals [28], dynamic brain networks [29] and even to diagnose diseases such as Parkinson’s [30].

Here, we apply the RQA to study the consistency of the cooperative display in the swallow-tailed manakin *Chiroxiphia caudata* (Passeriformes: Pipridae), a lekking species in the Neotropics [31]. Courtship displays in this species are made up of several independent and stereotyped movements performed consecutively (see video in [22]). Besides similarities with congeners in display structure, the group (or court) size of cooperative swallow-tailed manakin males is notably different because they gather in groups of two to six individuals [22,32], while males of lance-tailed manakin (*C. lanceolata* [33]) and long-tailed manakin [8] display in pairs. Swallow-tailed manakin displays begin with the dominant male making strong high-frequency vocalizations, directing other males and the female to the display perch [34]. Vocalizations are usually performed by more than one male, resulting in a duet or chorus [34]. The display itself involves repeated and coordinated movements: males perch in a row next to the female and then perform a circular display flight, jumping and vocalizing towards her [22,32]. Males flap their wings quickly in a hovering flight in front of the female, during which they slightly tilt their body and head down. After this short flight, each male returns to the end of the row, thus giving way to the next male [22,32]. All these movements compose the ‘cartwheel flight display’ [22,32]. Males accelerate and increase their flight frequency over time until a different vocalization from the dominant male determines the end of the cooperative display [22,32,34]. Along with this vocalization, the dominant hovers towards the other males, then lands on a higher branch, while the other individuals leave the perch [22,32]. After the cartwheel display, copulation occurs 66.7% [32] to 90% [22] of the time if immediately followed by the solo display of the alpha male.

In this study, we aimed to characterize the time series of the swallow-tailed manakin’s display and assess its consistency. We hypothesized that (i) display consistency would vary with group size, as changes in the number of individuals could influence dance performance, and (2) females prefer more consistent displays because these could indicate good qualities in males that could be inherited by their offspring. To test these hypotheses, we generated a time series of their cartwheel displays and quantified their frequency along with five key RQA metrics: RR, DET, LAM, MCEntr and recurrence radius. Consistency was characterized by an increase in RR and DET and a decrease in MCEntr. We predicted that (i) display characteristics would vary with male group size, such that RQA metrics and display frequency would correlate with the number of males, and (ii) female visitation rates (number of visits to the display perch) and copulation rates (with the dominant male) would be higher for more consistent displays and larger groups.

## 2. Methods

### 2.1. Study area

The study was carried out in two Brazilian protected areas, in approximately 4 km^2^ at Mananciais da Serra (48°59′ W, 25°29′ S), Piraquara, PR, between 2015 and 2019 and in approximately 2 km^2^ at Salto Morato Nature Reserve (48°15′ W, 25°10′ S), Guaraqueçaba, PR, in 2018 and 2019. Vegetation cover in both areas ranges from Montana Dense to Mixed Rainforests [35,36].

### 2.2. Capture and marking

We searched for perches daily, between September and March, in places where we heard males on display or producing alert vocalizations. After identifying the display perch, we captured birds using mist nets three times a week during the breeding period of the swallow-tailed manakin (October to February; [32,37]). When captured, we banded males and females with a numbered metal provided by the Brazilian agency CEMAVE/ICMBio (banding permit #1195110, project #3871) and three coloured plastic bands for later identification.

### 2.3. Collecting display behaviour data

We monitored all display perches used by males, either the traditional or accessories (or secondary) perches [32]. We video-recorded perches using Sony HDR-CX230 digital cameras placed at 5 m for approximately five consecutive hours autonomously. The cameras were positioned to optimize image capture at the same height as the perch and with its full length visible so that all dancing individuals and the female could be captured at the same time. The recordings were made four times a week, sampling different courts whenever possible.

From each recording, we identified the start and end times of all displays and the number and identity of participating individuals. We considered that a display was finished when males performed the *keekeekee* element of the cartwheel display [22] or when displaying males or the female left the perch. We also recorded the occurrence of female visits and copulations after the display. We used the MotionMeerkat programme (v. 2.0.5; [38]) to detect at which point in the recordings an individual had appeared on the perch, and then the Kdenlive^®^ programme to cut and create new files with video frames where the displays were detected.

**Figure 1.**
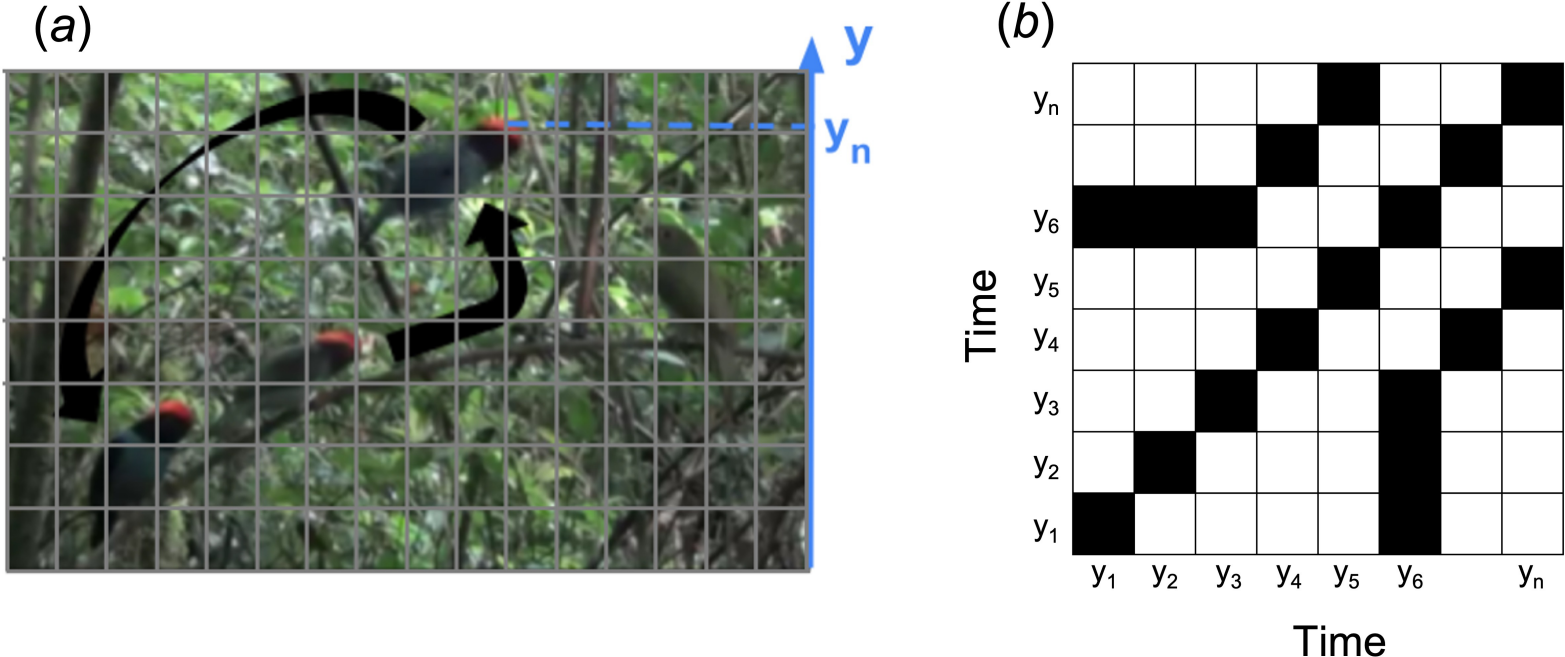
Illustration of the method used to quantify the display of the swallow-tailed manakin. (*a*) The background image shows a female on the right and three males on the display. Black arrows mean the direction of movement performed in the cartwheel display. The grey grid illustrates how the programme divides the frame into quadrants to track the red in the males’ heads. The dashed blue line highlights the vertical position (*y*-axis) of the male who is closest to the female. (*b*) Illustration of a recurrence plot; *y_n_* means the vertical position at time *n*, and filled cells indicate the coincident vertical positions of the males in the display.

After selecting each display in the videos, we produced a script in the Python programming language (v. 3.6 [39]), using the packages *OpenCV* [40], *math* [39], *NumPy* [41], *SciPy* [42] and *Matplotlib* [43] to track the individuals in the display flights and to extract their position. This script scans and tracks pixels based on colour, which we set as ‘red’ (ranging from tones [150, 0, 0] to [255, 100, 100] RGB designation) as a reference for males’ heads. The programme tracks each frame of the video file in quadrants and records the vertical position of each male’s head (figure 1*a*). The male position is only recorded in a given quadrant when a percentage of that quadrant is covered by the chosen colour. To determine which percentage to use, we tested the values of 30, 40, 50, 60 and 70% of the covered quadrant and made an ‘operating characteristic curve’-like by plotting the number of errors against each percentage used to find out which value had the best performance. We found that the 40% value was the best fit.

In order to improve code performance and considering that 30 frames per second is much higher than the necessary sampling, a downsampling was done to 15 frames per second with a Python script (v. 3.6; [39]). The window values are the vertical positions (*y*-axis, figure 1*a*) of the red markers from the tracker algorithm when a male reaches the edge of the dance, i.e. when he is positioned horizontally closer to the female or, when the female is absent, when he is further to the left or right in the dance, depending on the direction of the dance (figure 1*a*). This position is independent of male identity. Then, when another male has his horizontal position at the edge of the dance, the programme stops tracking the previous male and records the vertical position of this sequential male. This process is repeated throughout the whole video recording. The male identity is not saved, and the final time series represents the males’ vertical movement over the display.

We calculated the frequency and produced the recurrent plots using vertical time series. The frequency can be understood as the number of cartwheel flights per second since each vertical oscillation represents one flight. For this procedure, we created a script using the Python packages *math* [39], *NumPy* [41], *SciPy* [42] and *Matplotlib* [43].

### 2.4. Recurrence quantification analysis

RQA is made upon the recurrence matrix (or recurrence plots, figure 1*b*) [25], which is a square matrix of binary states (0 or 1). The rows and columns have the length of the time series, and each cell (*i*,*j*) of this matrix assumes the value 1 if the *i*th term of the time series has the same value (with a precision of +*r*, a parameter of the analysis; see below) as the *j*th term; otherwise, it assumes 0 (figure 1*b*). Thus, the matrix always has its main diagonal equal to 1 (because each cell will represent a recurrent point when compared with itself) and will be symmetric (since if *i* is recurrent to *j*, *j* is to *i*).

We followed the procedure of Prado *et al*. [26] to set the precision parameter *r*. The procedure uses the maximum entropy principle [44], where the computation of the recurrence entropy is maximized as a function of *r*. We extracted five metrics of the RQA: (i) RR is the percentage of cells in the recurrence matrix that are recurrent. It decreases as the number of different registered heights in a time series increases. So then, an interpretation of RR can be the variability of elements (here, heights) in the time series. (ii) DET is the percentage of cells in the recurrence matrix that form diagonal lines, indicating the percentage of the time series that has parts of sequential data repeated over the series. DET indicates how predictable the future motion is as a function of the past motion in the display. (iii) LAM is the percentage of sequential cells in the recurrence matrix with no variation (horizontal lines or vertical lines, if you look at the upper or lower triangular part of the matrix, respectively, figure 1*b*). LAM indicates the percentage of time series composed of stationary moments, i.e. how long will males remain immobile (or with only horizontal variation). (iv) MCEntr measures the Shannon entropy of sub-matrices of size 2 × 2, which are randomly sampled from the recurrence matrix. The probabilities *P*_i_ of each microstate can be obtained directly from the corresponding recurrence plot, and the entropy is calculated by Shannon entropy. The maximization of entropy ensures that most of the recurrence information from the system will be inserted in the recurrence matrix. Even the smallest changes in the dynamics slightly change the probabilities and, consequently, the entropy. This is a highly sensitive metric able to detect subtle differences between displays because it quantifies entropy in a finer timescale window [26]. (v) Recurrence radius (*r*), or optimum vicinity parameter, which is based on the entropy maximization [25,26,45]. If this measure varies with different time series, it means that these series have different general patterns. However, as we are analysing similar patterns of display (swallow-tailed manakin cooperative display recorded in the same position), it is expected that there will be no difference in this metric for this study. Observe that RR, DET and MCEntr compute the central idea of consistency presented here—the increase of the first two metrics and the decrease of the latter signify the increase in consistency.

To perform RQA, we use the ‘Julia’ programming language (v. 1.4.1 [46]), with the packages ‘DifferentialEquations.jl’ [47], ‘Random.jl’ [48], ‘LaTeXStrings.jl’ (Copyright © 2013−2014 by Steven G. Johnson), ‘Sundials.jl’ [47], ‘DelimitedFiles.jl’ [46], ‘Statistics.jl’ [48] and ‘DynamicalSystems.jl’ [49].

### 2.5. Female visitation and copulation rate

For each group of males, we identified the number of female visits and the number of copulations in all perches recorded. We considered one female visit each time a female appeared in the perch, attending or not a cooperative or solo display. We divided these numbers by the total recording times of each perch to calculate female visitation and copulation rates, respectively.

### 2.6. Statistical analyses

First, we tested data normality using the Shapiro–Wilk normality test from the *stats* package [50] and the *psych* package [51]. Because only DET had a normal distribution, we tested the Spearman correlation among the recurrence metrics (RR, DET, LAM, MCEntr and *r*) using the *psych* package [51].

We tested the relationship between the number of males present in the display (predictor variable) with cartwheel flight frequency (response variable) and metrics of RQA (RR, DET, LAM, MCEntr and *r*, response variables) using generalized linear mixed models (GLMM) with a Gamma distribution with a log link function in the *lme4* package [52]. We also used GLMMs to test the relationship between female visitation and copulation rates (response variables) with the number of males, metrics of RQA and cartwheel flight frequency (predictor variables). For the female visitation and copulation rates, we tested collinearity between predictors with the *VIF* R package [53], detecting high collinearity (VIF > 3.5) for DET and LAM when both variables were included in the models. Therefore, we excluded DET because it showed the highest VIF (>9). In all GLMMs, we included court identity as the random effect. Model fits were tested using the *performance* package [54]. We use the package *heatmaply* [55] for the correlation plot. To produce all the LMM graphs, we used the *ggeffects* [56] and *ggplot2* [57] packages. All statistical analyses and graphs were made in the R 4.1.0 programme [50].

## 3. Results

**Figure 2.**
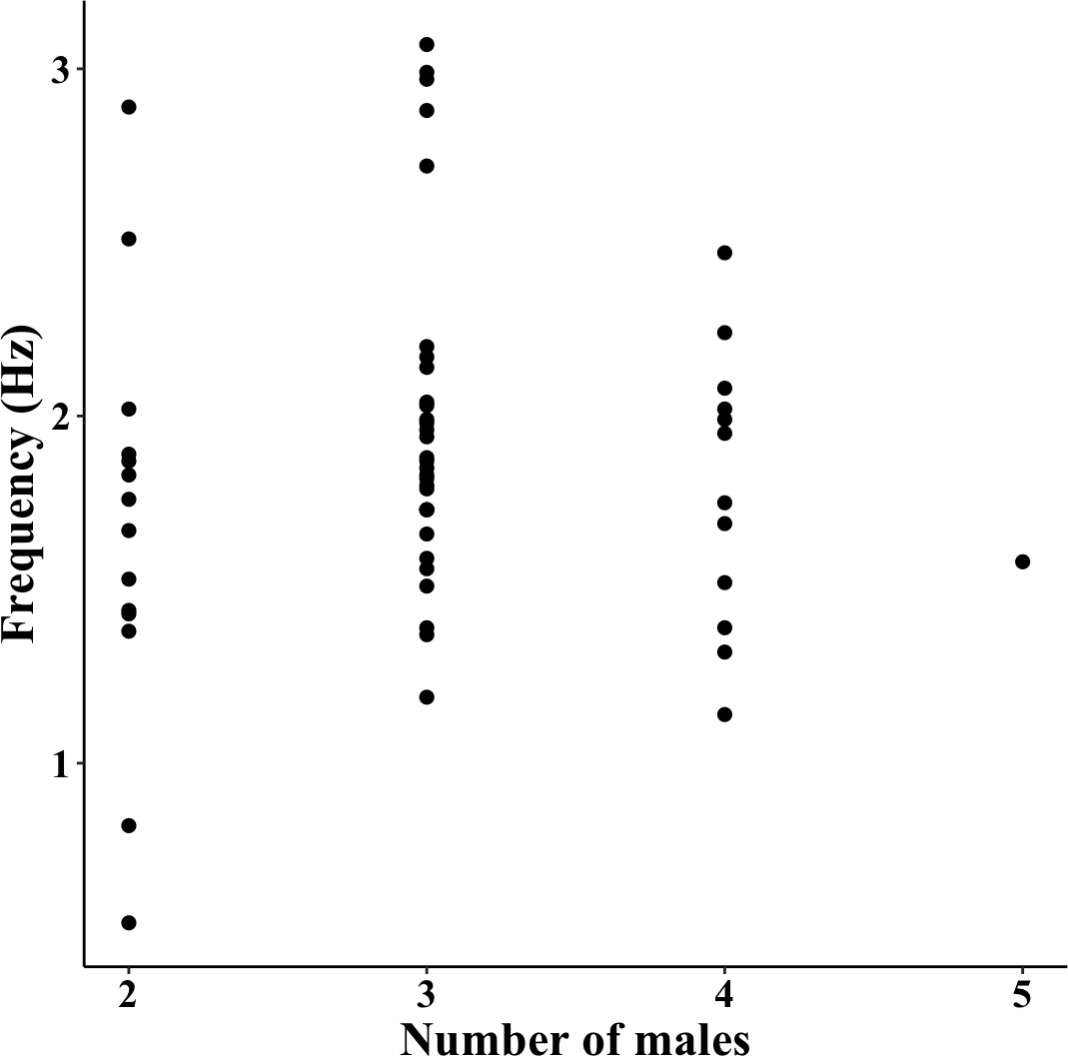
The number of males in the display in relation to the mean frequency of the cartwheel flight (coefficient of variation = 26.9). The frequency did not change with the number of males.

**Figure 3.**
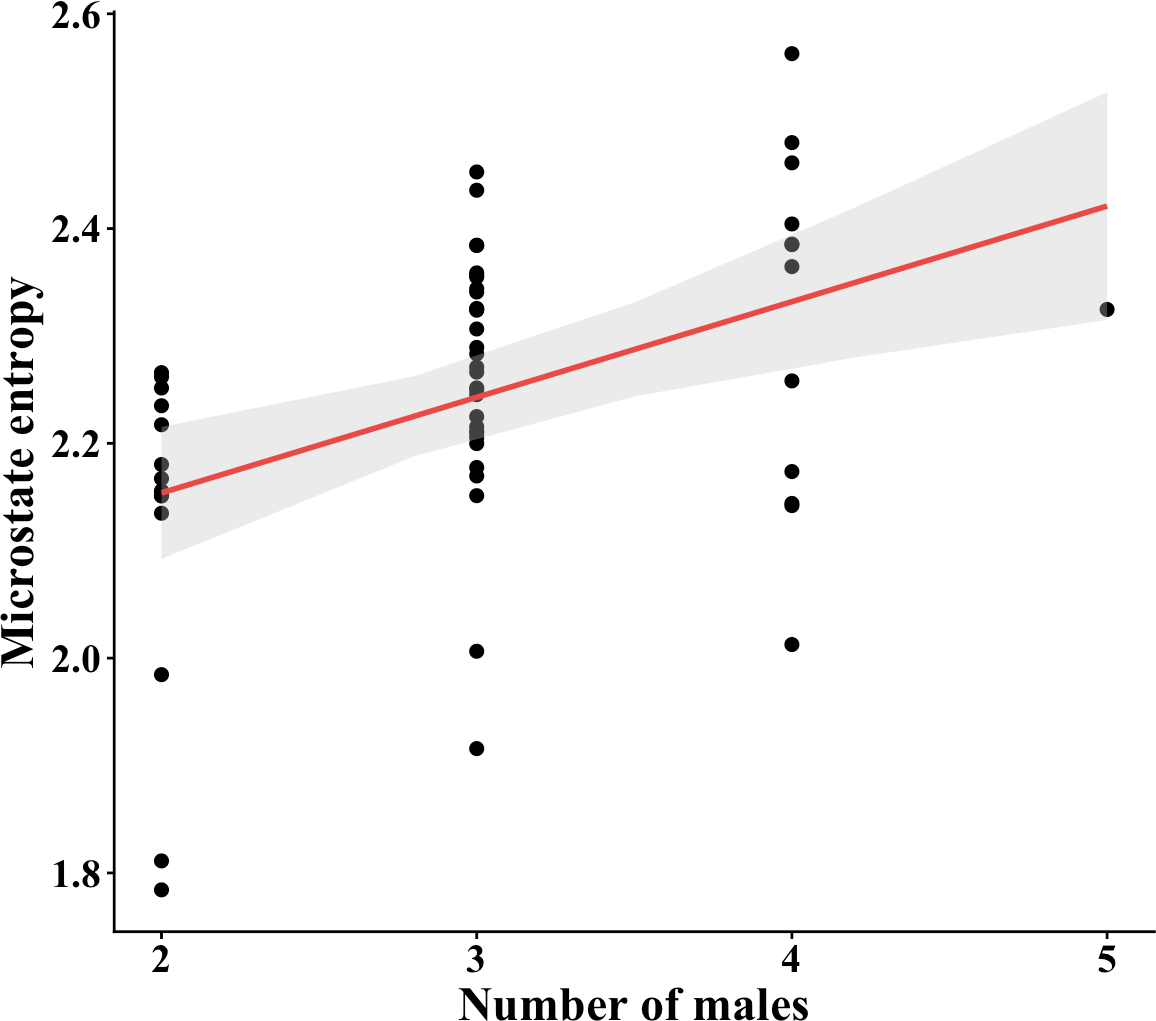
Relationship between the microstate entropy (MCEntr) and the number of males present in the display. The red line indicates the relationship, and the grey area means the 95% confidence interval. MCEntr was the only RQA metric related to the number of males, indicating that the variation in the number of males only produces subtle changes in male movements.

We analysed 58 displays of 6 courts (9.67 ± 4.96 displays/court), in which 2.96 ± 0.54 males participated per display. The average cartwheel flight frequency per display was 1.89 ± 0.50 Hz (min = 0.54 Hz, max = 3.07 Hz). We did not find a relationship between the number of males and the cartwheel flight frequency (β ± s.e. = 0.01 ± 0.06, χ^2^ = 0.04, *p* = 0.84, figure 2). Among all RQA variables, only DET was correlated with LAM (0.83) and MCEntr (−0.57) (electronic supplementary material, figure S1). We found that MCEntr was related to the number of males (β ± s.e. = 0.04 ± 0.01, χ^2^ = 7.76, *p* < 0.01, figure 3); the greater the number of males in the display, the greater the MCEntr. However, the number of males was not significant in the GLMM of the other RQA variables (RR: 0.02 ± 0.02, χ^2^ = 0.97, *p* = 0.32; DET: −0.01 ± 0.01, χ^2^ = 1.42, *p* = 0.23; LAM: −0.003 ± 0.008, χ^2^ = 0.12, *p* = 0.72; and *r*: −0.002 ± 0.01, χ^2^ = 0.03, *p* = 0.87).

**Figure 4.**
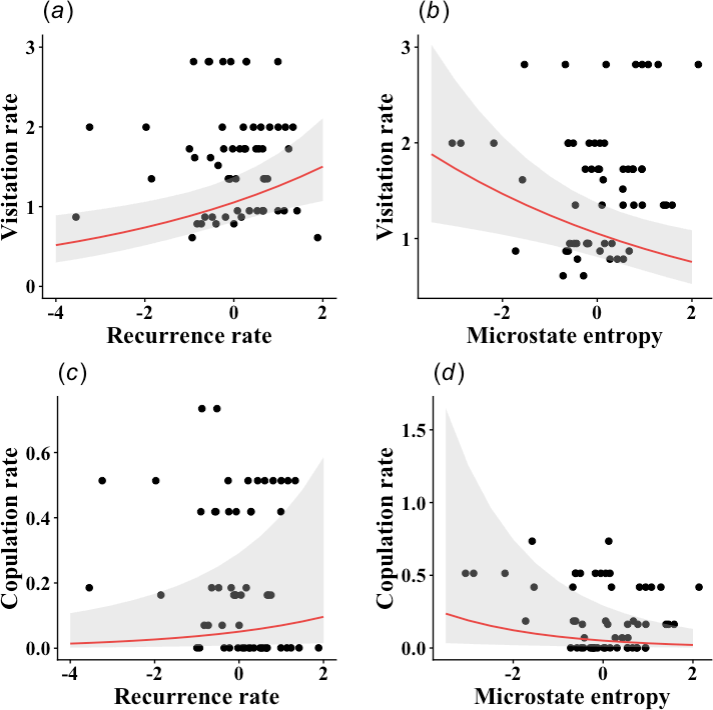
Relationship between female visitation rate (visits/h) and copulation rates (copulations/h) and the significant RQA variables (*a*) recurrence rate (RR), and (*b*) microstate entropy (MCEntr). The grey area means the 95% confidence interval. Female visitation and copulation rates increase as the display consistency increases (higher RR and lower MCEntr). The *x*-axis is centred at zero and scaled by the standard deviation.

**Figure 5.**
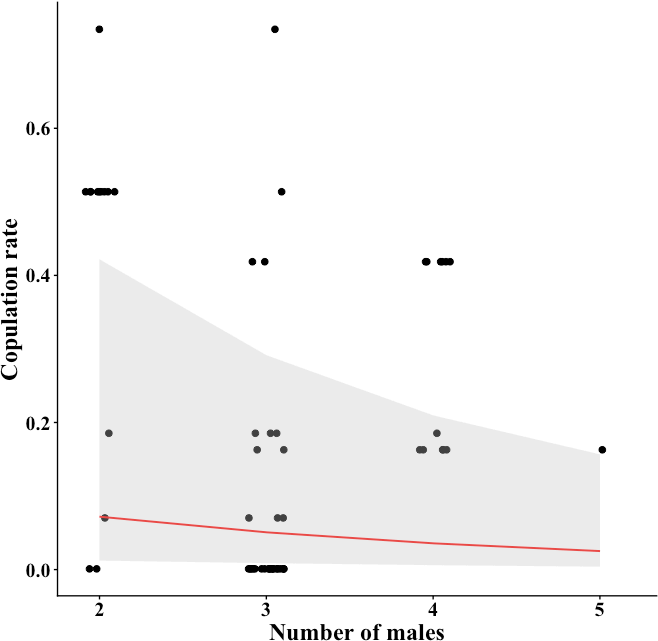
Relationship between the number of males present in the display and copulation rates (copulations/h). The grey area means the 95% confidence interval. Copulation rates decrease as the number of males increases. Data points are displayed with horizontal jitter to avoid overplotting.

We found no relationship between female visitation and copulation rates with cartwheel flight frequency, LAM and *r* (table 1, electronic supplementary material, figure S2), while both rates were related to RR and MCEntr (table 1). Visitation and copulation rates increased with RR and decreased with MCEntr (figure 4). The number of males in the display did not influence the female visitation rate but was negatively related to the copulation rate, i.e. copulations were more likely when the male group was smaller (table 1, figure 5).

**Table 1.** Result of the GLMM analyses relating female visitation and copulation rates with number of males in the display, cartwheel frequency and the RQA variables, recurrence rate (RR), laminarity (LAM), microstate entropy (MCEntr) and recurrence radius (*r*).

response variable	predictor	estimate (s.e.)	d.f.	χ^2^	*p*
female visitation rate^a^	intercept	0.05 (0.33)			
	number of males	−0.04 (0.04)	1	0.95	0.33
	cartwheel frequency	0.02 (0.04)	1	0.14	0.70
	RR	0.18 (0.05)	1	11.34	<0.01^c^
	LAM	−0.08 (0.04)	1	3.30	0.07
	MCEntr	−0.16 (0.05)	1	9.63	<0.01^c^
	*r*	−0.01 (0.03)	1	0.09	0.76
copulation rate^b^	intercept	−2.98 (1.75)			
	number of males	−0.25 (0.10)	1	5.89	0.01^c^
	cartwheel frequency	0.07 (0.13)	1	0.25	0.62
	RR	0.32 (0.16)	1	4.13	0.04^c^
	LAM	−0.06 (0.13)	1	0.19	0.66
	MCEntr	−0.44 (0.16)	1	7.58	<0.01^c^
	*r*	0.02 (0.10)	1	0.05	0.82

^a^ Variance ± s.d. of the random effect: 0.09 ± 0.31.

^b^ Variance ± s.d. of the random effect: 4.50 ± 2.12.

^c^ Significant *p* values.

## 4. Discussion

Here, we characterized and evaluated the consistency of the courtship displays of the swallow-tailed manakin in relation to the number of displaying males and female choice. We quantified the display frequency and five RQA metrics: RR, DET, MCEntr, LAM and recurrence radius. We defined display consistency as an increase in RR and DET, coupled with a decrease in MCEntr. Our results revealed that while display frequency did not vary significantly with group size, larger groups exhibited higher MCEntr and fewer copulations, suggesting that increasing male numbers leads to less consistent displays and reduced reproductive success. Furthermore, females visited and copulated more frequently with males performing highly consistent displays (i.e. those with higher RRs and lower MCEntr).

Our results indicate that the number of males does not influence the general pattern of the dances, which varied from two to five participating males. The analysed temporal series was measured in terms of the vertical movement of ‘red’ pixels (colour present in the head of males, as explained in §2), regardless of the individual identity. All analysed metrics of these series were not sensitive to the number of males, except the MCEntr, which captures only subtle changes in the time series. Then, having the same general pattern means that a sequence made by, for example, four individuals in a given time period is the same as two sequences of two individuals in the same time period. These results suggest that swallow-tailed manakin males cooperatively reduce individual effort in the courtship display by maintaining the same overall pattern of the display with fewer energetic demands. For instance, in a five-individual group, a male makes fewer cartwheel flights and spends a longer time in the perch than if he was displayed in a duo or trio; yet, the collective performance remains unchanged. To our knowledge, this finding represents the first evidence of energy-saving cooperation in avian courtship displays, a phenomenon previously undocumented despite its prevalence in other social contexts. For example, migratory birds flying in flocks reduce individual energy expenditure [58], and chimney swifts use coordinated circular flight patterns as they approach their roost to minimize collision effort [59]. Similarly, cliff swallows in tandem flights prioritize manoeuvrability over energy conservation [60]. The parallels across these systems highlight that effort reduction or agility enhancement is a fundamental benefit of cooperative flight in birds.

Even though we found evidence for a benefit on energy saving for the cooperative display, alternative explanations in our study species may also rely on female preference for larger aggregations [61] or kin selection [62], in which helpers are genetically related to the individual who copulates and thus have their fitness increased indirectly. However, evidence for kin selection in Pipridae is still conflicting, with studies corroborating [63] or not [64–66] this hypothesis. Furthermore, considering that subordinates are part of the court but rarely copulate, cooperation on display can improve their manoeuvring skills and facilitate reaching the alpha position in the future (e.g. [67]). In general, despite the increased complexity and disorder in larger groups, there are beneficial reasons for the aggregation in several taxa [68]. In fiddler crabs (Crustacea: Ocypodidae), for example, despite the presence of females being the trigger for synchrony between males, the density of males also interferes with the synchronization [69]. Similarly, individuals of Carolina chickadees (*Poecile carolinensis*) in larger groups used calls with greater complexity than in smaller groups [70].

We found that RR and MCEntr were related to both female visitation and copulation rates. Despite the MCEntr indicating only subtle variations in the dance performance, the RR reflects the overall degree of variability in flight heights throughout the dance. Therefore, when taken together, these results indicate that females select consistent displays for visiting and copulating. In lance-tailed manakins, consistency is also important to attract the female audience; when they attend the dance, the display is more consistent [9]. This relationship between consistency and audience suggests that consistency is important for female choice, as the coordination between alpha and beta can sign cognitive and motor abilities and, thus, male quality. Likewise, repetitive signalling is important for mate choice [71], and this might also be the case for the swallow-tailed manakins. In this way, displaying males can signal their quality to the female, as they manage to maintain the repeatability of the signal despite the energetic costs [72]. While honest signalling provides a compelling framework for these results, alternative mechanisms such as sensory drive [73] or Fisherian runaway selection (reviewed in [74]) could also contribute to the observed pattern, favouring consistent displays as either an efficient stimulus or an arbitrary trait that co-evolves with a female preference. Currently, evidence for these alternatives in manakins remains limited (e.g. [75–77]), and our data do not allow us to distinguish between these possibilities. Therefore, we cannot yet rule out that a combination of mechanisms, rather than a single process, underpins the importance of display consistency in swallow-tailed manakin courtship.

As the number of males in the dance increases, the entropy also increases, and the copulation rate reduces. Thus, there are subtle differences in the movements performed by the males in the display that matter for female choice. Other Pipridae species are known for males performing rapid movements with their wings, producing mechanical sounds with specialized structures [78,79]. In the club-winged manakin (*Machaeropterus deliciosus*), the display is extremely fast and can only be perceived by human eyes in video recordings at 250 frames per second [78]. These movements may be short and discreet. They can also be associated with female choice, as seen in the Golden-collared manakin (*Manacus vitellinus*), where females prefer males performing flights tens of milliseconds faster than others [80]. Our results show that female choice for finer movements also occurs in the swallow-tailed manakin, and our study opens a new avenue for understanding how sexual selection operates in this species.

Our findings demonstrate that while the general pattern of the swallow-tailed manakin’s dance remains stable across group sizes, female choice is sensitive to subtle variations in display consistency—specifically, higher RRs and lower MCEntr. This suggests that females evaluate fine-scale temporal precision rather than the overall display pattern, potentially using consistency as a proxy for male quality (e.g. motor skill or endurance). Notably, the increase in entropy with group size, despite minimal changes in the dance’s structure, implies that males in larger groups face challenges in maintaining individual precision, which may explain the observed decline in copulation success. These results align with studies in other Pipridae, where females select mates based on nuanced performance traits (e.g. millisecond-scale differences in golden-collared manakins; [80]).

Our study adds to the body of literature showing that RQA is a powerful tool for evaluating animal display (e.g. [24]) because it detects patterns at different signal levels, both in the overall series and in discreet details [25,81]. Although it has recently been used to characterize crab displays [24], this is the first study to use the RQA to study courtship in birds, being a promising approach to better understanding animal behavioural patterns. Our findings indicate that consistency should be a crucial aspect for male organization during the dance and for female choice, and future studies of sexual selection could incorporate this axis of variation by quantifying repeatability, predictability and timing of signal emission, in addition to traditional metrics of ordering and structure.

## Data Availability

The data and codes are provided as electronic supplementary materials [82].
